# Theoretically Unveiling the Factors That Control Activated Ion Mobility in Lithium-Based Polymerized Ionic Liquids and Glasses

**DOI:** 10.1021/acscentsci.6c00171

**Published:** 2026-05-04

**Authors:** Ankita Das, Harmandeep Singh, Catalin Gainaru, Alexei P. Sokolov, Kenneth S. Schweizer

**Affiliations:** † Department of Materials Science, 242176 University of Illinois, Urbana−Champaign, Urbana, Illinois 61801, United States; ‡ Materials Research Laboratory, 4292 University of Illinois, Urbana−Champaign, Urbana, Illinois 61801, United States; § Department of Physics and Astronomy, University of Tennessee, Knoxville, Tennessee 37996, United States; ⊥ Chemical Sciences Division, 6146 Oak Ridge National Laboratory, Oak Ridge, Tennessee 37831, United States; ¶ Department of Chemistry, University of Tennessee, Knoxville, Tennessee 37996, United States; ∥ Department of Chemistry, University of Illinois, Urbana−Champaign, Urbana, Illinois 61801, United States; ◆ Department of Chemical & Biomolecular Engineering, University of Illinois, Urbana−Champaign, Urbana, Illinois 61801, United States

## Abstract

Understanding the activated transport of ions in neat polymeric liquids and glasses is a fundamental scientific problem in materials, polymer, and physical chemistry, and it is highly relevant for diverse energy storage applications. Of special interest are cation-based single ion conductors, “polymerized ionic liquids” (PolyILs). We combine three recent advances in microscopic statistical mechanical theory for multiscale structure, ion hopping, and polymer segmental dynamics to address how structural correlations, thermodynamic state, and anion–cation association strength determine the temperature-dependent activation barrier for ion transport and its coupling with polymer motion. The key is a new mapping of molecular complexity to a coarse grained model that encodes the chemically specific glass transition temperature, *T*_*g*_ , polymer persistence length, vitrification density, and anion–cation attraction strength. Quantitative comparisons with the mobile Li ion relaxation times of PolyILs with a Triflouromethane Sulfonimide (TFSI) anion, and predictions for a theoretically designed model with a lower *T*_*g*_ and Coulomb attraction, are presented. New core results include a critical Coulomb strength being required for the onset of ion activated motion and a master curve over 15 decades of hopping rate based on a temperature-dependent effective anion–cation association energy that encodes variations of polymer flexibility, Coulomb strength, temperature, and density. The strong role of correlated polymer dynamical fluctuations in facilitating ion hopping is established.

## Graphical abstract

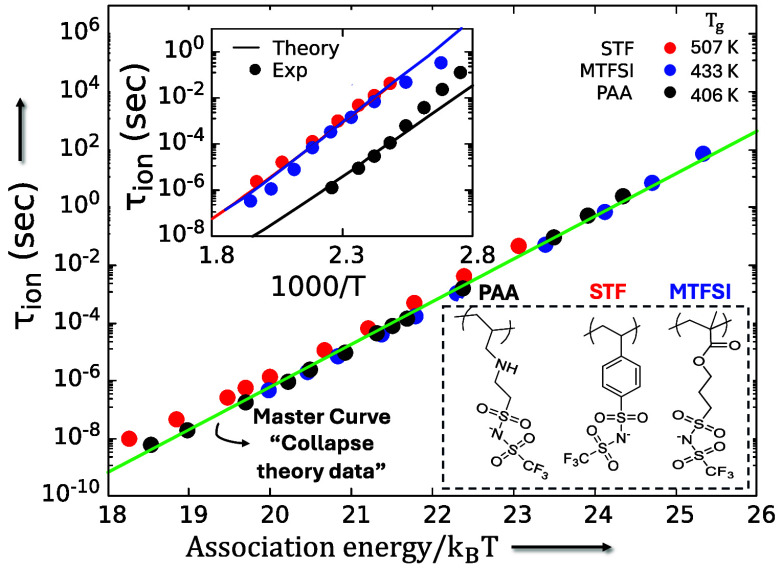

## Introduction

Polymerized ionic liquids (PolyILs) and glasses are single ion conductors that exhibit rich and fascinating physical behaviors with potentially high relevance for energy storage in general and solid state batteries in particular.^1–12^ However, their low room temperature conductivity has thwarted widespread applications. A vexing issue is that strong anion–cation Coulomb attraction and the coupling of ion hopping with slower polymer segmental relaxation conspire to induce large activation barriers for charge transport. Fundamental theoretical understanding of the diverse structural and dynamical behaviors of PolyILs remains a grand challenge.

Very recently we developed a suite of first of their kind microscopic statistical mechanical theories for the multiscale structure,^13^ coupled segmental and ion activated dynamics,^14–16^ and structural relaxation and the glass transition temperature *T*_*g*_ ^16^ for PolyILs within a modestly coarse grained polymer physics framework. The predictions have been explored via numerical model calculations. Significant understanding of how the ion-monomer size ratio, polymer backbone stiffness, and Coulomb interaction strength determine structure^13^ and *T*_*g*_ of a large experimental data set^16^ has been achieved. Most recently, a predictive theory of cation hopping in deeply supercooled liquids and glasses has been formulated.^15^ Major novel findings include the dominance of Coulomb cage scale correlations and attractions in determining ion hopping rates, an activation barrier only emerges above a critical threshold strength of Coulomb attraction that far exceeds thermal energy, and the barrier grows nonlinearly with electrostatic attraction due to structural and charge correlation effects.

The above advances set the stage for the present work which fully integrates all theoretical methods and formulates a new physical chemistry and polymer science motivated mapping to incorporate chemical complexity in the coarse grained PolyIL model (PolyIL Model, Theories, and Chemical Complexity Mapping). This advance allows quantitative predictions to be made for experimental systems, here focused on three well characterized Li-TFSI based PolyILs (Experimental Results for Li-PolyILs). The ideas are quantitatively contrasted with the experiments in Ion Relaxation Time Results: Theory vs Experiment and provide a unified framework to understand them. The theories are then employed as an exploratory discovery tool, first in Predictions for a Lower *T*_*g*_ Theoretical Material which studies a “theoretically designed” model PolyIL with a lower *T*_*g*_ and Coulomb attraction strength than for existing Li-TFSI PolyILs. Sensitivity to Cation–Anion Association Energy and Universal Master Curve and New Mechanistic Theoretical Insights present broader predictions for how multiple chemical and physical variables affect the ion hopping rate, discusses possible new synthetic routes to superionic conduction, establishes the role of correlated polymer dynamical motion on ion hopping, and unveils a surprising universality that encompasses chemical variations of polymer flexibility, Coulomb attraction strength, temperature, and density relevant to the design of high conductivity materials. To the best of our knowledge, our work is the first attempt to use statistical mechanical theory to address PolyIL activated dynamics in the low temperature, high density, and long time scale range relevant to experiments,^1,2,8,11,17–21^ beyond time scales accessible to simulation.^9,10,22–39^ The paper concludes in Open Issues and Future Directions with a discussion of open questions and future directions. Supporting Information (SI) includes some details of the experimental materials, theoretical modeling, plus additional figures that enrich the main messages of the article.

## Experimental Results for Li-PolyILs

We consider the three chemically diverse Li-PolyILs in Figure 1d that all have the *same* Li-TFSI anion–cation moiety on the side chain: STF (poly­(styrene trifluoromethane sulfonimide)), MTFSI (poly­(methacrylate trifluoromethane sulfonimide)), PAA (poly­(allylamine trifluoromethane sulfonimide)).^8,11,16,40^ We will analyze the most recently published ion relaxation time data^41^ where *T*_*g*_ equals 507 K (STF), 433 K (MTFSI), 406 K (PAA). The Li activated relaxation time, *τ*_*ion*_, is extracted via analysis^11,41^ of the frequency dependent conductivity function, *σ*_*C*_(ω). The corresponding DC ionic conductivity, *σ*_*DC*_ , obeys the classic BNN relation^42^ corresponding to *Tσ*_*DC*_ ∝ τ_*ion*_^‑1^ with a nearly universal prefactor weakly dependent on temperature (Figure S1). Details of the synthesis and characterization of these PolyILs have been thoroughly described in prior articles, and is summarized with references in the SI.

**Figure 1:**
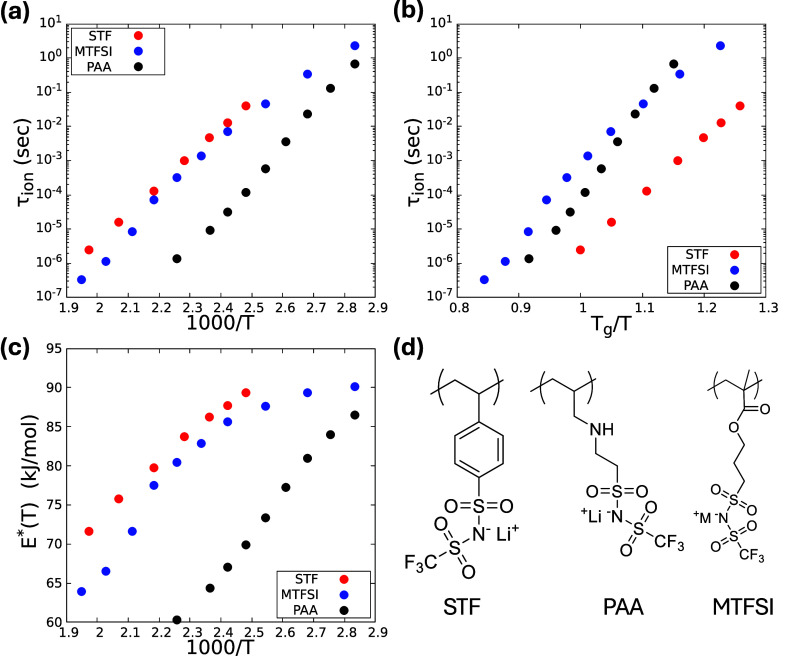
Experimental data for the Li-TFSI based PolyILs STF, PAA, and MTFSI. Ion relaxation time (s) plotted versus inverse temperature (Kelvin) (a) and in a dimensionless *T*_*g*_-scaled format (b). (c) The extracted physical barrier in kJ/mol as a function of inverse temperature, which at *T*_*g*_ are 71, 81, and 69 for STF, MTFSI, and PAA, respectively. (d) Chemical structures of the polymers studied. Adapted from ref 41. Copyright 2025 American Chemical Society.

Figure 1 presents *τ*_*ion*_ data as a function of inverse temperature (Figure 1a) and its *T*_*g*_-scaled analog (Figure 1b), which covers ∼7 decades from ∼300 *ns* to ∼5 *s* spanning the supercooled liquid and quenched glass regimes. Its relative ordering with polymer chemistry changes (partially inverts) if compared at fixed temperature versus at the polymer alpha time (*τ*_*α*_) isochronal state of fixed $\frac{T_{g}}{T}$ . Figure 1b shows that at *T*_*g*_, ion relaxation is strongly decoupled (per the classic Angell analysis for ionic glasses^43^) from structural relaxation (*τ*_*α*_(*T*_*g*_) ≈ 100*s*) by ∼5 to 8 decades. This is reflected in the roughly *apparent* Arrhenius growth of *τ*_*ion*_ with cooling, to within modest deviations. Such large decoupling implies low ion “steepness indices”^43^ at *T*_*g*_ defined as $m_{ion}\equiv \frac{d}{d\left(\frac{T_{g}}{T}\right)}\log\tau_{ion}\left(T_{g}\right)\approx 16-25$ . Figure 1c shows the effective *physical* activation barrier as a function of inverse temperature employing the now well-established relation^41^ $E^{*}\left(T\right)=k_{B}T\ln\frac{\tau_{ion}\left(T\right)}{\tau_{0}}$ , with a *fixed* and physical time prefactor of τ_0_ = 0.1 *ps*. The barrier growth with cooling proves the relaxation process is *not* literally Arrhenius. Figure S1 demonstrates the very close connection between *τ*_*ion*_ and the conductivity.

The *τ*_*ion*_ variations reflect chemical differences such as polymer backbone stiffness (expected to correlate with *T*_*g*_ per homopolymers^44–46^ ) and side chain structure which modify interchain packing and potentially the cation–anion association energy. Presumably modest differences in the density–temperature relationship at 1 atm as encoded in the equation of state (EOS) and dielectric properties are also relevant. The data in Figure 1 is employed below to render the statistical mechanical theories quantitatively relevant to the *τ*_*ion*_(*T*) measurements based on a novel mapping of PolyILs to a trained coarse grained model.

## PolyIL Model, Theories, and Chemical Complexity Mapping

All technical details of the adopted coarse grained model and theories have been exhaustively discussed in prior publications,^13,15,16^ and thus only a physical overview is given below. We then formulate new ideas for mapping PolyILs onto the polymer physics model along with descriptions of the temperature dependent density and dielectric constant.

### Coarse Grained Model, PolyIL Packing Structure, and Dynamical Theories

The coarse grained model^13–16^ of a 1:1 PolyIL is illustrated in the middle panel of Figure 2. Polymers consist of tangent semiflexible connected chains (persistence length *l*_*p*_) of negatively charged spheres (monomers) of hard core diameter σ. Mobile cations are smaller positively charged spheres of diameter *d*. These length scales enter as two key dimensionless ratios: the size asymmetry *d*/σ (∼0.17 for^47^ Li), and polymer backbone stiffness via a local aspect ratio *l*_*p*_/σ. Monomers and mobile ions interact via hard core repulsions plus effectively screened Coulomb interactions of known range.^48,49^ Coulomb strength is quantified by the *absolute value* of the anion–cation attraction energy at contact, *ε*_*mp*_(*T*) ≡ *e* ^2^/(*d*_*mp*_ *ϵ*_*d*_(*T*)), where $d_{mp}\equiv \frac{d+\sigma }{2}$ and *ϵ*_*d*_(*T*) is the temperature dependent dielectric constant discussed below; we adopt units where 4*πϵ*_0_ ≡ 1. For typical PolyILs, Coulomb attractions are very strong, *βε*_*mp*_ (*T*) ≫ 1, where β ≡ (*k*_*B*_ *T*)^−1^ is the inverse thermal energy. The thermodynamic state is set by temperature, and the dimensionless melt density or packing fraction φ­(*T*) that grows with cooling at fixed pressure.

**Figure 2:**
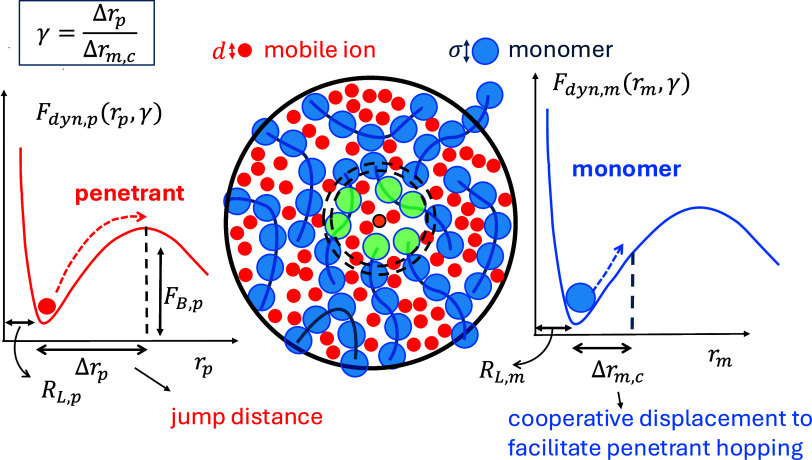
Elements of the PolyIL model and dynamical theories employed. Middle: schematic of the polymer physics model of a dense liquid of spherical charged monomers (blue circles) of diameter σ and oppositely charged small mobile ions (red circles) of diameter *d*. Left and right: schematic of two-step coupled ion and segmental activated motion controlled by their respective dynamic free energies in self-consistent collective hopping theory (SCCHT).^15,50–54^ Reproduced with permission from ref 15. Copyright 2025 American Institute of Physics.

Given the molecular interactions and thermodynamic state parameters, all intermolecular pair structural correlations (ion–ion, ion–monomer, monomer–monomer) in real space, *g*_*ij*_(*r*), and static partial structure factors in Fourier wavevector space, *S*_*ij*_(*k*), are computed using PRISM integral equation theory.^13^ This serves as the critical input to quantify dynamic constraints in the *spatially resolved* coupled dynamic free energies (Figure 2) for predicting correlated activated motion of ions and polymer segments in the self-consistent cooperative hopping theory (SCCHT)^15^ and elastically collective nonlinear Langevin equation (ECNLE)^16^ theory. Technical details, some comparisons to experiment, and core predictions for PolyIL melts and glasses based on numerical model calculations are documented in prior articles.^13–16^

Figure 2 illustrates the physical ideas of SCCHT. The faster process corresponds to ion hopping (barrier crossing) from a localized state of a spatially resolved dynamic free energy, *F*_*dyn*__,*p*_, that depends on the instantaneous ion (*r*_*p*_) and monomer (*r*_*m*_) displacements. It is a priori constructed from knowledge of all microstructural correlations determined from PRISM theory.^13,55,56^ Ion hopping is facilitated by small in-cage monomer dynamical fluctuations controlled by their own dynamic free energy, *F*_*dyn*__,*m*_, which also depends on monomer and ion displacements. Coupled activated motion is quantified at the stochastic trajectory level by the parameter γ, defined as the ratio of the ion jump distance from its localized position to its barrier, to the corresponding in-cage correlated monomer displacement. The mean ion hopping time is computed by enforcing *temporal* dynamic self-consistency of these two events, which determines γ. For small Li cations the longer range collective elastic barrier associated with hopping is negligible, and the local Coulomb cage barrier dominates. After ions have hopped and begun to diffuse, they are an ergodic fluid (decoupling) from the monomer perspective, and the second slower monomer activated structural relaxation occurs on a modified dynamic free energy that includes all Coulomb-mediated microstructural correlations.^16,54^ Structural relaxation within the ECNLE theory framework^16^ involves a total barrier with coupled local cage and collective elastic contributions. All mean activated relaxation times (*τ*_*ion*_(*T*), *τ*_*α*_(*T*)) are computed using Kramers theory where the most important quantity is the microscopic activation barrier.^50,51^ Barriers grow with cooling and density due to strengthening of dynamical constraints associated with changes of PolyIL structure, primarily via the ion–monomer and monomer–monomer correlations on the Coulomb cage scale.

We now consider the first new goal which is to address the missing atomistic Angstrom-scale chemical detail in the coarse grained model via mapping and model training ideas. These allow determination of the core chemistry and temperature dependent parameters: $\frac{l_{p}}{\sigma },\varepsilon_{mp},\epsilon_{d}\left(T\right),\varphi \left(T\right)$ , which allows testable dynamical predictions for real PolyILs to be made.

### Multistep Mapping of Chemical Complexities

We propose a coordinated 4-step mapping strategy based on *T*_*g*_ as a reference state where ion and monomer dynamics are strongly decoupled. This choice is motivated by our prior findings^13,15,16^ there is essentially *no* plasticization of *τ*_*α*_ and *T*_*g*_ since the Li volume fraction is tiny, and the precise anion–cation (large) Coulomb attraction strength has little effect on *T*_*g*_. These simplicities imply understanding *T*_*g*_ of Li-PolyILs is to leading order a problem of the corresponding neutral homopolymer melt, for which the persistence length and packing fraction are the key variables.^16,44–46^ There are 4 steps of the mapping.

**Step 1.** The theoretical prediction for each PolyIL *T*_*g*_ is calibrated to agree with experiment, as previously discussed.^8,11,16,40^ This is achieved by varying the semiflexible chain backbone stiffness and reduced density to reproduce the experimental *T*_*g*_ values using ECNLE theory to compute *τ*_*α*_(*T*), thereby yielding a *polymer-specific* connection between *T*_*g*_, φ­(*T*_*g*_) ≡ *φ*_*g*_, and $\frac{l_{p}}{\sigma }$ . An aspect ratio of $\frac{l_{p}}{\sigma }=2.7$ is assigned to what we believe is the stiffest polymer, STF.^57^ Theoretical reproduction of its *T*_*g*_ = 507*K* requires *φ*_*g*_ = 0.63. This mapping process is repeated for the other PolyILs (see Figure S2 and Table S1), yielding *φ*_*g*_ = 0.58, 0.56 and $\frac{l_{p}}{\sigma }=1.69,2.0$ for PAA and MTFSI, respectively. Higher local aspect ratio implies higher *T*_*g*_ and lower density at *T*_*g*_, as expected.^16,44^

Importantly, the above deduced range of $\frac{l_{p}}{\sigma }$ ∼1.7–2.7 are consistent with simulations^58^ that employed the same semiflexible polymer model and established what aspect ratios correspond to the physical behavior of dozens of real homopolymer melts. These simulations found^58^ the Kuhn length to monomer diameter ratios are $\frac{l_{K}}{\sigma }\approx 2-4.$ For the tangent semiflexible chain Koyama model^46^ $\frac{l_{p}}{\sigma }=0.5\left(1+\frac{l_{K}}{\sigma }\right)$ , implying $\frac{l_{p}}{\sigma }=1.5-2.5$ , consistent with our analysis.

**Step 2.** A priori quantitative prediction of the anion–cation association energy for real PolyILs based on a coarse grained model is impossible. To address this, we build on Step 1 and determine the dimensionless Coulomb attraction strength at contact at the *single* state point of *T*_*g*_, $\beta_{g}\varepsilon_{mp}\left(T_{g}\right)\equiv \frac{e^{2}}{\left[d_{mp}\epsilon_{d}\left(T_{g}\right)k_{B}T_{g}\right]}$ , by requiring SCCHT theory reproduces the experimental *τ*_*ion*_(*T*_*g*_) in Figure 1. This exercise gives *β*_*g*_ *ε*_*mp*_(*T*_*g*_) = 20.0, 22.3, 21.5 for STF, MTFSI, PAA, respectively; importantly, these are reasonable magnitudes.^59^ The corresponding contact association energies $\varepsilon_{mp}\left(T_{g}\right)=\frac{e^{2}}{\left[d_{mp}\epsilon_{d}\left(T_{g}\right)\right]}$ are 84, 80, 73 kJ/mol for STF, MTFSI, PAA, respectively, which are close presumably because of their common Li-TFSI functional group. The determination of these *β*_*g*_ *ε*_*mp*_(*T*_*g*_) values is deeply coupled to the Step 1 analysis since within the combined structural and dynamical theories they depend on *T*_*g*_, $\frac{l_{p}}{\sigma },\varphi_{g}$ . Hence, the mapped coarse grained model implicitly encodes Angstrom-scale information in *ε*_*mp*_(*T*_*g*_) since backbone stiffness and side chain structure impact density, dielectric constant, packing correlations, and *T*_*g*_. The precise values of the “bare” Coulomb attraction strength, $\frac{e^{2}}{\left[d_{mp}\right]}$ , cannot be deduced since *ϵ*_*d*_(*T*_*g*_) is unknown.

**Step 3.** To *predict* ion relaxation times at *all* other temperatures requires the isobaric temperature-dependent packing fraction. As in prior work,^14–16^ since PolyIL EOS data is absent, based on the Step 1 deduced *φ*_*g*_ we compute φ­(*T*) as its linear change with temperature using reasonable thermal expansion coefficients of α = 8 × 10^–4^ *K* ^–1^ (liquid), 4 × 10^–4^ *K* ^–1^ (glass). Below and previously^14,15^ we verified the precise numbers have minor effects for ion dynamics.

**Step 4.** The temperature-dependent dielectric constant enters in the dimensionless anion–cation attraction strength parameter, *βε*_*mp*_(*T*) ∝ ϵ_*d*_^‑1^(*T*), at the fundamental level of screened Coulomb effective potentials employed in the PRISM theory of PolyIL structure.^13,55^ For physical consistency with this, plus our adoption of a model that averages over (sub)­picosecond time scales (THz frequencies), the relevant dielectric constant should be the higher frequency component that reflects equilibrated small scale fast motions *before* ions become transiently localized, and *long before* ion hopping and structural relaxation occurs. This dielectric constant has contributions from fast electronic (polarizability, refractive index) and vibrational degrees of freedom, which generically *grow* with cooling and densification. Other short time and small distance contributions such as in cage “ion rattling” motion may also be relevant.^35^

We adopt a minimalist mathematical model for *ϵ*_*d*_(*T*_*g*_) which increases with cooling and is consistent with the SCCHT physics and our parameter mappings at *T*_*g*_. It is characterized by a single *constant* positive exponent “y” in the range [0,1]:

1 $$\epsilon_{d}\left(T\right)=\epsilon_{d}\left(T_{g}\right){\left[\frac{T_{g}}{T}\right]}^{1-y}$$

2 $$\beta \varepsilon_{mp}\left(T\right)=\beta_{g}\varepsilon_{mp}\left(T_{g}\right){\left[\frac{T_{g}}{T}\right]}^{y}=\frac{e^{2}}{k_{B}\epsilon_{d}\left(T_{g}\right)T_{g}d_{mp}}{\left[\frac{T_{g}}{T}\right]}^{y}$$

This functional form in eq 1 is not fundamental. Note that y = 0 yields ideal Curie law behavior, and y = 1 gives a temperature independent dielectric constant; we expect real PolyILs have an intermediate value y. Over the typical experimental temperature range, the change of the high frequency dielectric constant is not large. But, crucially, we have shown^15^ that the ion barrier is *very* sensitive to small changes of *βε*_*mp*_(*T*), a central theme of our present study. This temperature-sensitivity can be quantified by – *d* *ϵ*_*d*_(*T*)/*dT*|_*T*_*g*__ = (1 – *y*)*ϵ*_*d*_(*T*_*g*_) *T*_*g*_^‑1^; an estimate using y = 0.5, our PolyIL *T*_*g*_’s, and *ϵ*_*d*_(*T*_*g*_) ≈ 3, yields – *d* *ϵ*_*d*_(*T*)/*dT*|_*T*_*g*__ ≈ 0.003–0.0045 *K* ^–1^. Such values are comparable (but larger as expected) than the temperature sensitivity of the optical dielectric constants of homopolymer melts, e.g., *d* *ϵ*_*d*_(*T*)/*dT*|_*T*_*g*__ ≈ 0.001–0.002 *K* ^–1^ for polystyrene and polypropylene.^60^ Future experiments that measure *ϵ*_*d*_(*T*) in the THz regime would be very valuable. Finally, the high frequency dielectric constant growth with cooling implies *reduction* of the *ε*_*mp*_(*T*) that enters the effective interaction potentials at lower temperatures. Hence, the predicted physical ion barrier growth with cooling derives from the enhanced Coulomb structural correlations as *βε*_*mp*_(*T*) and density increase upon lowering temperature at fixed pressure.

## Ion Relaxation Time Results: Theory vs Experiment

### Dielectric Constant Temperature Dependence and Bare Association Energies

The final required element is the exponent “y” in Eq ([Disp-formula eq1]). We adopt a simple approach that employs a *polymer and temperature independent constant* value. It is determined by asking what *single* value of y best reproduces the experimental *τ*_*ion*_ of *all* 3 PolyILs at a *single* reference temperature, *T*_*ref*_. Success is *not* guaranteed.

Figure 3a shows the results for *T*_*ref*_ = 400*K*. We find y = 0.6 is best, and nontrivially works well; this conclusion is robust to other choices of *T*_*ref*_ (see Figures S3 and S4). The results also show the precise temperature dependence of *ϵ*_*d*_(*T*) has a large effect on *τ*_*ion*_(*T*); increasing y yields a stronger separation of the different PolyIL ion relaxation times. All calculations below use y = 0.6, and thus $\beta \varepsilon_{mp}\left(T\right)=\beta_{g}\varepsilon_{mp}\left(T_{g}\right){\left[\frac{T_{g}}{T}\right]}^{0.6}$ . In reality, y could be nonuniversal and weakly temperature-dependent since eq 1 is not exact, but such an elaboration is not pursued here.

**Figure 3:**
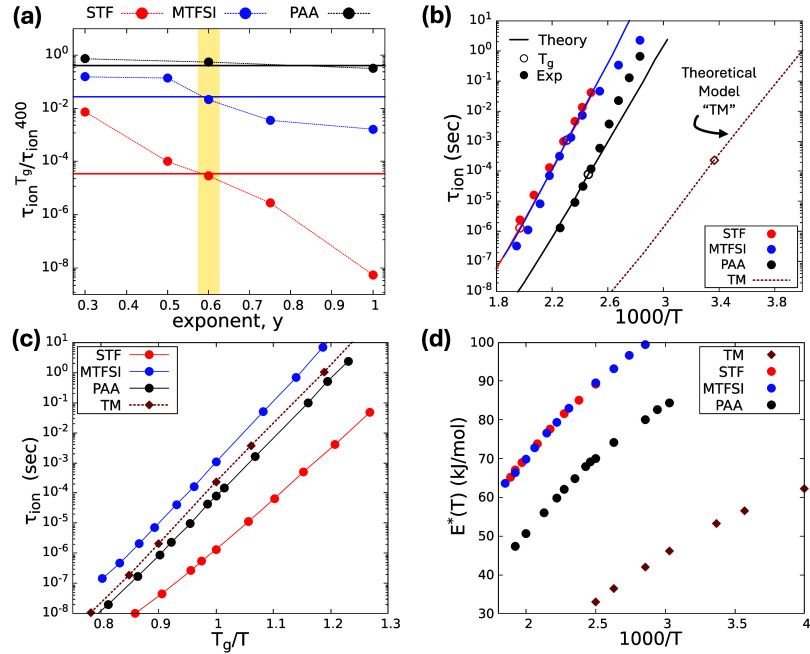
(a) Horizontal lines are the experimental ratio of $\frac{\tau_{ion}\left(T_{g}\right)}{\tau_{ion}\left(T=400\;K\right)}$ from Figure 1. The points are the corresponding theory calculations for PAA, MTFSI, and STF for different exponents *y*, *all based on* requiring the theory to reproduce *τ*_*ion*_(*T*_*g*_) of each PolyIL. The value *y* = 0.6 simultaneously matches the experimental results for all systems. (b) Temperature dependent theoretical *τ*_*ion*_ using *y* = 0.6 (solid curves) versus inverse temperature (Kelvin) compared to the data of Figure 1 (points) and for the theoretical model (TM) discussed in the text. (c) Same theory results (now shown as points with smooth lines as guides to the eye) as in panel (b) but as a function of inverse temperature scaled by *T*_*g*_. (d) Theoretical ion physical barrier $E^{*}\left(T\right)=k_{B}T\;\ln\frac{\tau_{ion}\left(T\right)}{\tau_{0}}$ vs inverse temperature with τ_0_ = 0.1 ps.

From the above analysis, the bare anion–cation association energy obeys *e* ^2^/*d*_*mp*_ ∝ *ε*_*mp*_(*T*_*g*_)/*T*_*g*_^0.4^. Taking STF as a reference state, and given $\frac{\varepsilon_{mp}}{\varepsilon_{mp,STF}}=\left[\beta_{g}\varepsilon_{mp}\left(T_{g}\right)/\beta_{g,STF}\varepsilon_{mp,STF}\left(T_{g}\right)\right]{\left(\frac{T_{g}}{507}\right)}^{0.6}$ , we deduce the bare association energy is a factor of 1.01 and 0.94 different for MTFSI and PAA, respectively. Thus, STF and MTFSI are effectively identical, and PAA almost so. This seems intuitive since all three PolyILs involve Li-TFSI.

### Full Temperature Dependence of Ion Relaxation Time: Theory vs Experiment

Figures 3b-3d presents *no* adjustable parameter calculations of all properties for the 3 PolyILs in Figures 1a-1c using y = 0.6. The extent of *τ*_*ion*_ growth with cooling for different PolyILs is well captured, as is the partial inversion of relaxation time ordering (Figure 1b vs 1c) when plotted versus *T*_*g*_/*T* in contrast to 1000/*T*. The high sensitivity of the predicted temperature dependences of *τ*_*ion*_(*T*) to the dielectric constant exponent is demonstrated in Figures S3 and S4 for smaller and larger values of y = 0.3 and y = 1. These results do not capture as well the temperature and relative PolyIL chemistry differences of *τ*_*ion*_ of the experimental data compared to when y = 0.6. This is explicitly shown in Figure S5 by overlaying theory and experiment for *τ*_*ion*_(*T*) when y = 0.3, 0.6, 1.

For y = 0.6 (and other values) the predicted functional forms of *τ*_*ion*_(*T*) are nearly *apparent* Arrhenius, with “strong” liquid like small steepness indices *m*_*ion*_ ≈ 17, 19, 20 for STF, MTFSI, PAA, in accord with the experimental estimates in Experimental Results for Li-PolyILs. The apparent Arrhenius behavior agrees very well with the STF data (including the apparent activation energy), while MTFSI and PAA exhibit small deviations likely reflecting polymer-specific deviations from our modeling of the EOS, aspect ratio, and/or temperature dependence of the dielectric constant.

It may seem puzzling that *τ*_*ion*_(*T*) appear visually Arrhenius-like given the input to the microscopic dynamical theory involves the *temperature-dependent* density, dielectric constant, and correlated multiscale structure. Physically, this is understandable as a consequence of the strong decoupling of the much faster ion hopping process from structural relaxation. Very importantly, the view that the ion relaxation process is literally Arrhenius is an illusion for the experimental PolyILs analyzed here.^41^ Figure 3d shows the theoretical physical barrier computed using $E^{*}\left(T\right)=k_{B}T\ln\frac{\tau_{ion}\left(T\right)}{\tau_{0}}$ and τ_0_ = 0.1 *ps* grow significantly with cooling due to temperature-dependent changes of density, dimensionless Coulomb strength, and packing structure. The functional form and rate of growth is in semiquantitative agreement with experiment (Figure 1d). For example, at 1000/T = 2.6, all E* values fall in a common range of ∼ 65–90 kJ/mol. The physical barriers are plotted in the *T*_*g*_-scaled representation in Figure S6. We note that whether PolyILs under external geometric or structural (e.g., microphase-separated) confinement follow literal or only apparent Arrhenius behavior is an open question.

## Predictions for a Lower *T*_*g*_ Theoretical Material

The experimental Li-PolyILs analyzed have the same TFSI anion, and rather large values of *T*_*g*_ and *τ*_*ion*_ To expand the generality of our work, we employ the theory as an exploratory tool by constructing a hypothetical “theoretical model” (TM) PolyIL with a near room temperature *T*_*g*_ and an anion that *more weakly* attracts Li than does TFSI. Specifically, we set *T*_*g*_ = 297 *K*, which is achieved for $\frac{l_{p}}{\sigma }=1.37$ and *φ*_*g*_ = 0.63 (Figure S2). The corresponding bare Coulomb attraction energy is chosen to be 20% smaller than the reference STF value of the prior section. Based on SCCHT and these parameters, the dimensionless Coulomb attraction energy at *T*_*g*_ is computed to be *β*_*g*_ *ε*_*mp*_(*T*_*g*_) = 21.6. The theory then predicts *τ*_*ion*_ ≈ 10^–4^ *s* at room temperature, much smaller than for STF, MTFSI, PAA.

Construction of the TM is also motivated by PolyIL experiments^61^ with boron-based anions (more charge delocalized) attached to a lower *T*_*g*_ poly­(methacrylate) backbone.^58^ Three polymers with especially bulky side groups have a low *T*_*g*_ ≈ 211–243 *K* and *σ*_*DC*_ ≈ 10^–6^ - 10^–4^ *S cm* ^–1^ at *T* ≈ 300 *K* which is ∼8–10 decades higher than that of Li-TFSI based PAA.^11,41^ We do not aim to quantitatively model these boron-based systems here, but rather they serve as additional inspiration for studying the TM model.

Theoretical predictions for *τ*_*ion*_(*T*) of the TM are shown in Figures 3b−3d. An enormous speeding up of hopping is predicted under isothermal conditions, with a complex relationship to the 3 Li-TFSI PolyILs under constant $\frac{T_{g}}{T}$ conditions due to competing effects. A high sensitivity of the *apparent* Arrhenius temperature dependences of *τ*_*ion*_(*T*) to the dielectric constant exponent is again found (Figure S3).

## Sensitivity to Cation–Anion Association Energy and Universal Master Curve

### Tuning the Anion–Cation Association Energy

An essential finding of our previous model calculations was the ion hopping barrier can be greatly reduced by only modest reduction of the Coulomb association energy.^15^ We elaborate on this important behavior for the 3 mapped PolyIL models and TM by computing how *τ*_*ion*_(*T*) changes if *only* the bare *ε*_*mp*_ is reduced by factors of 0.9, 0.8, 0.7 with *no change* of any other parameter (*fixed* aspect ratio, temperature, density, dielectric constant). The results in Figure 4a show ion hopping is strongly accelerated in a nearly exponential manner by up to 6–8 decades in a manner weakly dependent on PolyIL chemistry. Such large effects reflect our core prediction:^15^ the Li ion barrier vanishes below a threshold Coulomb attraction strength that is not small, *β*_*g*_ *ε*_*mp*_ (*T*_*g*_) ≫ 1. This suggests modest modifications of Li-PolyIL structure associated with the polymer backbone, the non-TFSI part of the side chain, use of non-TFSI anions with weaker association energy, and/or attaching bulkier groups near the anion could induce large conductivity increases. For example, for the TM the ion hopping rate is predicted to be enhanced by another ∼5 decades (*τ*_*ion*_ ≈ 10^–9^ *s*) near room temperature if *ε*_*mp*_ → 0.8*ε*_*mp*_.

**Figure 4:**
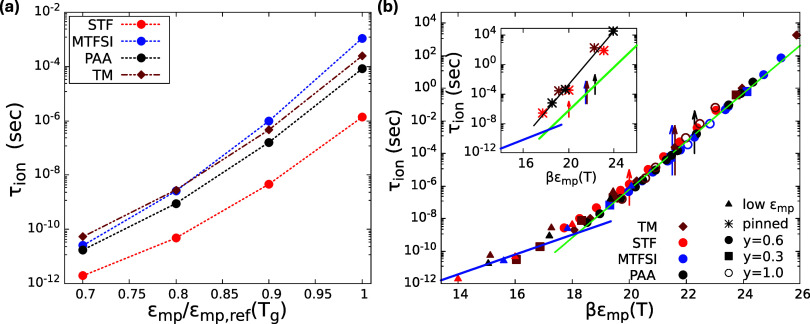
(a) Predicted ion relaxation times for PolyIL models with the *same* aspect ratios and packing fractions at *T*_*g*_ as STF, MTFSI, PAA, and TM in Figure 3b, c where *ε*_*mp*_ (expressed in terms of *ε*_*mp,ref*_(*T*_*g*_) as the reference state) is reduced by a factor of 0.9, 0.8, and 0.7. (b) A well collapsed master curve is predicted based on all the *τ*_*ion*_(*T*) calculations in Figures 3a, d, S3, and S4, including those with variable dielectric constant exponents *y*, plus the additional calculations in panel (a), all plotted as a function of the temperature and polymer chemistry dependent dimensionless Coulomb attraction strength. The data collapse into roughly two regimes with a smooth crossover as illustrated by the green and blue lines. The arrows indicate each PolyIL calculation with *y* = 0.6 at *T*_*g*_. Inset: asterisks are the corresponding results if the polymer is dynamically pinned at its localization length when the ion hops as discussed in Role of Facilitating Polymer Dynamic Fluctuations.

### Universality, Master Curve, and Superionic Conductivity

The *τ*_*ion*_(*T*) results are numerically obtained from a rather elaborate self-consistent microscopic theory that depends, even for the coarse grained model adopted, on: (i) polymer stiffness, (ii) ion-monomer size ratio, (iii) density, (iv) temperature, (v) bare association energy, (vi) temperature-dependent dielectric constant, (vii) *T*_*g*_, and (viii) the trajectory level coupling parameter (γ in Figure 2). But might the theory predict an emergent simplicity, perhaps as a consequence of strong Coulomb attractions and decoupling?

We explore this intriguing question motivated by the possibility that *βε*_*mp*_(*T*) could be the relevant quantity *if* the chemical, vitrification, and thermodynamic parameters are related (as they must be in reality) via the correlated specification of aspect ratio, *T*_*g*_, and density at fixed pressure, per our mapping scheme. We plot in Figure 4b *all* the *τ*_*ion*_(*T*) calculations for y = 0.6, and also for y = 0.3 and 1, from Figures.[Fig fig3], 4a, S3, and S4 in a log–linear manner as a function of *βε*_*mp*_(*T*). This large computational data set covers 15 decades of *τ*_*ion*_ from 1000 s to 1 ps. The theory predicts a remarkable and surprisingly good collapse onto two linear roughly exponential branches, with vertical fluctuations of order one decade or less.

The higher relaxation time branch covers ∼ 9 decades down to ∼ 1 ns which may be the entire realizable range for experimental PolyILs. Notably, this large range is associated with a dimensionless Coulomb attraction strength that varies only over the modest range *βε*_*mp*_(*T*) ∼ 18–26, all corresponding to strong Coulomb attractions. This data is well represented by

3 $$\tau_{ion}\left(\beta \varepsilon_{mp}\left(T\right)\right)=1.25\;ps\times \exp\left[\left(3.4(\beta \varepsilon_{mp}\left(T\right)-16.15\right)\right]$$

There are three important features of eq 3. First, the predicted effective time prefactor of 1.25 ps is a physical value for a condensed phase barrier crossing event.^62^ Second, the origin and order of magnitude of the subtractive 16.15 factor *qualitatively* reflects the core SCCHT prediction that ion localization (temporally stable anion–cation associations) and activated hopping transport *only* occur for high enough Coulomb strength, the barrier threshold effect for Li.^15^ The latter implies *τ*_*ion*_ can be enormously reduced by a relatively small decrease of *βε*_*mp*_(*T*). We emphasize that eq 3 *only* applies to the upper branch of our numerical results which do *not* extend down to *βε*_*mp*_(*T*) = 16.15 Thus, the precise 16.15 number has no deep significance, and does *not* correspond to when the barrier vanishes (see below). Third, the numerical factor 3.4 has an intuitive physical basis per our prior calculations of the cation–anion pair correlation function.^13^ It closely agrees with^13^ the mean number of nearest neighbor anions solvating a Li ion in its Coulomb cage, *N*_*cage*_, and also with the *dimensionless* electrostatic cohesive energy,^63^ |*U*_*coh*_|/*ε*_*mp*_, for $\frac{d}{\sigma }=0.17.$ Even for Li-based materials, size ratio can be reduced by employing larger volume monomers, e.g., via bulkier side groups as in some boron-based PolyILs.^57^ The theory anticipates a significant enhancement of the ion hopping rate *even if* the strength of the bare Coulomb attraction remains unchanged since *N*_*cage*_ and |*U*_*coh*_|/*ε*_*mp*_ become smaller for geometric packing reasons as $\frac{d}{\sigma }$ decreases.

The extension of the first branch in Figure 4b down to *τ*_*ion*_ ≈ *ns* at *βε*_*mp*_ (*T*) ≈ 18 (physical barrier of ∼ 12 *k*_*B*_ *T*) has important implications for achieving room temperature superionic conduction. Consider the TM for which *τ*_*ion*_(*T* = *T*_*g*_ = 297*K*) ≈ 10^–4^ *s* with *βε*_*mp*_(*T*) = 21.6. If a chemical modification was made that reduced the bare association energy by only ∼ 17% so that *βε*_*mp*_(*T*) = 18, Figure 4b predicts the ion hopping rate increases by ∼4 decades to ∼10^8^ *s* ^–1^, approaching that required for superionic conductivity.

For small enough *βε*_*mp*_(*T*) there is a smooth crossover in Figure 4b to a second more roughly collapsed and weakly decreasing regime. This “bending over” and ultimate saturation of the numerical data *must occur* since SCCHT predicts the barrier *vanishes* as a threshold dimensionless Coulomb attraction strength is approached, here occurring at *βε*_*mp*_(*T*) ≈ 11–12. The shown guide to the eye line extends down to *τ*_*ion*_ ≈ *ps* at *βε*_*mp*_(*T*) ≈ 14 where the barrier is ≈ 3.4*k*_*B*_ *T*.

The above results fundamentally differ from highly phenomenological approaches such as the Anderson-Stuart (A-S)^64^ model which in our notation is *τ*_*ion*_(*T*) ≈ τ_0_ exp­(*b βε*_*mp*_(*T*)) with *b* an unknown constant. The barrier does not vanish (full ion delocalization) until *βε*_*mp*_(*T*) ≈ 0. In a more practical sense, to achieve *τ*_*ion*_(*T*) ≈ *ns* if τ_0_ = 0.1 *ps* requires *βε*_*mp*_(*T*) ≈ 9 assuming *b* = 1, or (as likely more appropriate) if *b* ≈ 3 per a realistic Li coordination number then the required *βε*_*mp*_(*T*) ≈ 3. Such weak Coulomb attractions seem chemically very unlikely or impossible, and are vastly smaller than *βε*_*mp*_(*T*) = 18 predicted by SCCHT due to the barrier threshold effect.

Finally, one might be surprised by the explicit disappearance of many physical factors in the master curve of Figure 4b. But one should not be misled by the fact that alternative models with y = 0.3 and y = 1, with different backbone stiffnesses, and/or different values of *T*_*g*_, all collapse to leading order onto the same master curve. This reflects their ${\beta \varepsilon }_{\mathrm{mp}}\left(T\right)=\beta_{g}\varepsilon_{mp}\left(T_{g}\right){\left(\frac{T_{g}}{T}\right)}^{1-y}$ values coincide at certain system-specific temperatures, not that they exhibit identical *τ*_*ion*_ at the same temperature (see Figures 3 and S3–S5). Most importantly, we emphasize that the material-specific values of *T*_*g*_, polymer aspect ratio, and density *do* enter our mapped calculations, albeit in a correlated manner. We now elaborate on this point, which reveals valuable new physical insights.

## New Mechanistic Theoretical Insights

To more deeply understand the predicted master curve in Figure 4b we perform three additional new theoretical analyzes that selectively remove key aspects of the theory and/or mapping. Such an exercise is not possible to perform in experiments nor in typical simulations.

### Role of Facilitating Polymer Dynamic Fluctuations

In SCCHT, the ion hopping event is strongly coupled at the trajectory level with *subcage* scale activated monomer motion (Figure 2). One can ask how important this physics is for *τ*_*ion*_(*T*) in an absolute sense, and also with regards to the existence of a master curve. To answer this question, the inset of Figure 4b shows representative calculations (asterisks) using y = 0.6 if we *pin by hand* the polymer monomers at their predicted small localization length corresponding to monomers only undergoing small amplitude harmonic vibrations. This removes the influence of the temporally self-consistent activated monomer dynamic fluctuations coupled to the ion barrier crossing event, the fundamental feature of SCCHT. The results demonstrate that the trends with *βε*_*mp*_(*T*) are qualitatively retained, but removing polymer dynamical fluctuations temporally correlated with ion hopping largely *destroys* the master curve. Moreover, the ion hopping rates are slower by ∼ 2–4 decades. Thus, the correlated small scale polymer motion of central importance to SCCHT is not a small effect, but rather strongly accelerates ion transport.

### Role of Correlation of Material Parameters in Real PolyILs

We now ask what is the consequence of *not* enforcing our multistep mapping to model isobaric PolyILs that casually relates the polymer aspect ratio, *T*_*g*_, and density? This is achieved by *independently* varying the packing fraction, aspect ratio, and dimensionless Coulomb attraction strength parameters (and hence *T*_*g*_ also). The representative results in Figure 5a show at fixed *βε*_*mp*_(*T*) that (a) *τ*_*ion*_ always grows (decreases) with packing fraction (aspect ratio), and (b) most importantly, the master curve collapse is destroyed demonstrating its existence is tied to mapping real PolyILs to the coarse grained model in an experimentally sensible manner.

**Figure 5:**
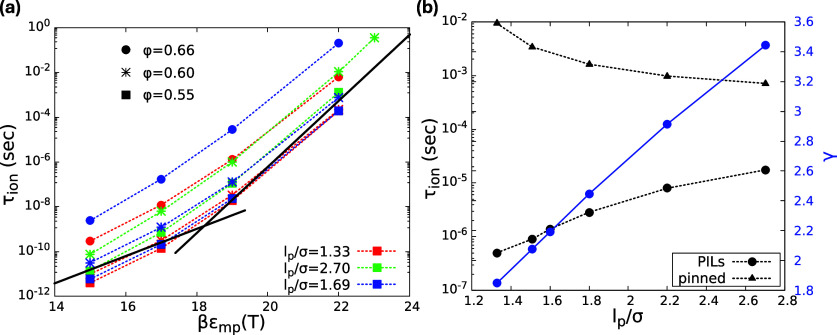
(a) Model ion relaxation time(s) as a function of βε_*mp*_(*T*) at *independently* chosen packing fractions (0.55, 0.60, 0.66) and aspect ratios (1.33, 1.69, 2.70) covering the range relevant for PAA, MTFSI, STF, and TM. The individual points of fixed color and symbol type roughly follow the shape of the master curve in Figure 4b but do not collapse well with vertical deviations of up to 3 decades. (b) Model calculations of *τ*_*ion*_ (black circles) if the aspect ratio is varied over a wide range at fixed φ = 0.60 and βε_*mp*_ = 20. Ions hop more slowly by a factor of up to ∼25 as the polymer backbone stiffens. The black triangles are the corresponding results if the polymers are dynamically pinned. Ion hopping is slower, although the effect of aspect ratio is reversed. The self-consistently determined trajectory displacement ratio coupling parameter (blue circles), γ, per Figure 2 that is relevant to the black circles grows with polymer stiffness (see right axis).

### Explicit Influence of Polymer Backbone Stiffness

Finally, we perform model calculations of *τ*_*ion*_(*T*) as a function solely of polymer aspect ratio at *fixed* packing fraction and dimensionless Coulomb attraction strength. The results in Figure 5b show *τ*_*ion*_ (black circles) monotonically increase with backbone stiffness. Physically, one can understand this as largely a consequence of an increase of φ/*φ*_*g*_, or equivalently $\frac{T_{g}}{T},$ for stiffer polymers suppresses the facilitating *in-cage* monomer motions and ion hopping rate. Also shown (black triangles) are the corresponding results if the polymers are dynamically pinned. Ion hopping slows down as in Figure 5a, although the effect of aspect ratio is *reversed*. The trajectory displacement ratio coupling parameter (defined in Figure 2), γ, relevant to the solid black circles is shown as the blue circles, and grows with polymer stiffness. The reason is activated ion and polymer displacements become gradually more decoupled as polymers stiffen and segmental motion slows, resulting in monomer in-cage dynamic fluctuations becoming *less* important. This trend is consistent with the tendency of the red and black points in Figure 5b to converge as aspect ratio grows since monomer pinning matters *less* as the trajectory displacement ratio increases.

Overall, the results in this section support the idea that in the collapsed master plot of Figure 4b the influence of φ, *T*_*g*_, and aspect ratio, though effectively ″hidden″, *are* present in an essential manner via Step 1 of the mapping scheme. These tightly inter-related variables also deeply impact the value of *β*_*g*_ *ε*_*mp*_(*T*_*g*_) via Step 2 of the mapping, and hence the key *x*-axis variable $\beta \varepsilon_{mp}\left(T\right)=\beta_{g}\varepsilon_{mp}\left(T_{g}\right){\left(\frac{T_{g}}{T}\right)}^{y}$ . The existence of the master curve collapse is thus sensitive to our construction of a trained coarse grained model where *T*_*g*_, aspect ratio, density, and reduced Coulomb strength at *T*_*g*_ are *all* necessarily fundamentally inter-related.

## Open Issues and Future Directions

We conclude by discussing outstanding scientific issues and future theoretical directions, first for Li-TFSI based PolyILs. The theory predicts nearly *apparent* Arrhenius behavior of *τ*_*ion*_. The experiments in Figure 1 often find this behavior, but mild non-Arrhenius variations with cooling can occur depending on polymer chemistry. The latter may be especially related to how the high frequency dielectric constant grows with temperature, and our simple model of eqs ([Disp-formula eq1]) and ([Disp-formula eq2]) requires a more quantitative system-specific basis. Making progress on this difficult theoretical problem would be facilitated by measurements of the temperature and PolyIL dependent high frequency dielectric constant. Another issue is we have focused on *τ*_*ion*_(*T*), but conductivity includes collective ion dynamical correlations, although these are small corrections on a logarithmic scale (Figure S1). A microscopic theory for such corrections (inverse Haven ratio) has been recently formulated and successfully tested against experiment,^14^ and can be integrated with the present approach. Anion-specific effects beyond TFSI can be absorbed into two effective parameters of the model. Charge delocalization associated with weaker anion–cation attraction (e.g., boron-based anions) enters via the reference value of the contact association energy, *ε*_*mp*_. The number of anion nearest neighbors of a tagged Li cation (Coulomb cage coordination number) is deeply related to PRISM theory predictions of the anion–cation pair correlation function, and can be tuned to model different chemistries via modification of the steric size ratio variable $\frac{d}{\sigma }$. Finally, we mention that the theoretical concepts developed here for ion motion in PolyILs are applicable beyond energy storage applications to polymer-based sensors, including thermal, strain, and humidity sensors, where transport–dynamics coupling governs sensitivity and response.^65^

An exciting near term new direction is to extend our suite of theories and proposed mapping ideas to PolyILs with larger cations such as Na and K . The experimental behavior is rich,^41^ and there are competing effects since larger cations experience weaker Coulomb attractions which can speed up hopping, but for geometric packing reasons have more Coulomb cage neighbors which slows down hopping. Our goal is to formulate and apply our theories and new mapping ideas in a *no* adjustable parameter manner to address the full range of larger mobile ions (e.g., Na, K, Cs, Imi, TFSI) in the *same* PolyIL materials studied here.

Concerning functional materials design, at present solvent-free Li-based PolyILs do not have sufficiently large conductivities at room temperature for practical battery applications. Our recent experimental work for Na and K cations^41^ did find 1–3 decades of conductivity enhancement for the same baseline polymer chemistries as Li. This is a nontrivial improvement, but the conductivities still fall short of those required for battery applications. Based on the theoretical insights gained in this work, we suggest superionic conductivity in small cation PolyILs may be achievable if the following 4 goals are realized by new polymer synthesis. (i) Replace TFSI with a more charge delocalized anion that forms weaker Coulomb associations. (ii) Design monomers with bulky globular-like side groups to sterically reduce the cation coordination number and Coulomb cohesive energy. (iii) Add passive highly polarizable groups to the monomer to increase the high frequency dielectric constant and further reduce the Coulomb association energy. (iv) Modify the monomer backbone chemistry to be more conformationally flexible in order to reduce *T*_*g*_ to around room temperature or below and enhance local polymer mobility.

Another exciting theoretical direction is the fundamental problem of dynamic decoupling of ion and polymer relaxation, per a Walden-like plot.^66–68^ The tools are in place to address this since we uniquely have integrated theories of structure (PRISM), ion hopping (SCCHT), and structural relaxation (ECNLE). Extension of our approach to non-TFSI anion based PolyILs with weaker Coulomb attractions and/or bulkier side groups with smaller ion-monomer size ratios, and to ion conducting polymers involving zwitterions,^20,69^ are also directions we are poised to study.

## Supplementary Material



## Notes

Selected details of new aspects of the theoretical modeling plus additional theoretical and experimental figures that enrich the main messages of the article; brief summary of synthesis and characterization details of the experimental PolyILs analyzed (PDF)
