# Supplementary material for: Theoretically Unveiling the Factors That Control Activated Ion Mobility in Lithium-Based Polymerized Ionic Liquids and Glasses
Source: ACS Cent Sci. 2026 May 4;12(5):658–68. doi: 10.1021/acscentsci.6c00171 (PMC13220211; doi:10.1021/acscentsci.6c00171)
Supplement: Supplementary file 1 [file oc6c00171_si_001.pdf]

Supplementary Information

Theoretically Unveiling the Factors That Control Activated Ion Mobility in Lithium-Based Polymerized Ionic Liquids and Glasses

Ankita Das[1,2], Harmandeep Singh[3], Catalin Gainaru[4], Alexei P. Sokolov[3-5] and Kenneth S. Schweizer[1,2,6,7,*]

[1]Department of Materials Science, University of Illinois, Urbana-Champaign, Urbana, Illinois 61801, United States

[2]Materials Research Laboratory, University of Illinois, Urbana-Champaign, Urbana, Illinois 61801, United States

[3]Department of Physics and Astronomy, University of Tennessee, Knoxville, Tennessee, 37996, United States

[4]Chemical Sciences Division, Oak Ridge National Laboratory, Oak Ridge, Tennessee, 37831, United States

[5]Department of Chemistry, University of Tennessee, Knoxville, Tennessee, 37996, United States

[6]Department of Chemistry, University of Illinois, Urbana-Champaign, Urbana, Illinois, 61801, United States

[7]Department of Chemical & Biomolecular Engineering, University of Illinois, Urbana-Champaign, Urbana, Illinois 61801, United States

*Corresponding author: kschweiz@illinois.edu

The synthesis and characterization details of all polymers discussed in the main text have been reported in prior articles. Here we briefly summarize essential elements. All polymers were precipitated from a solution and then additionally dialyzed to remove any remaining monomers. NMR spectra did not detect any monomer residues. The STF has[1] a MW~10,000 g/mol corresponding to a degree of polymerization (DP) ~30. MTFSI has[2] a MW ~ 25,000 g/mol and DP

~ 72. PAA without TFSI was bought from a vendor and had a MW~60,000 g/mol and DP ~ 1050. It was then functionalized with TFSI (~95% of monomers)[3]. Earlier work determined the MW dependence of T_g of the studied PolyILs which was found[4] not to change significantly above a MW~10k-20k, which is the situation for the data reproduced in Fig.1.

According to the generalized Nernst-Einstein (NE) equation[5], the conductivity is $\sigma_{DC} = H^{-1}e^2nD/k_BT$, where n is the ion number density, H^{-1} the inverse Haven ratio, and D the single ion diffusion constant associated with Coulomb cage escape. To leading order, $D \approx l^2/6\tau_{ion}$ where l is a small jump length distance required for Fickian diffusion. Fig.S1 plots the published experimental values[41] of $T\sigma_c$ versus the ion relaxation rate τ_{ion}^{-1} . One sees a good data collapse on to a power law with an exponent of nearly unity, with small deviations associated with material specific and weak temperature variation of l and H^{-1}. This supports our statement in section II that conductivity and τ_{ion} are nearly linearly related to within a close to universal constant of proportionality.

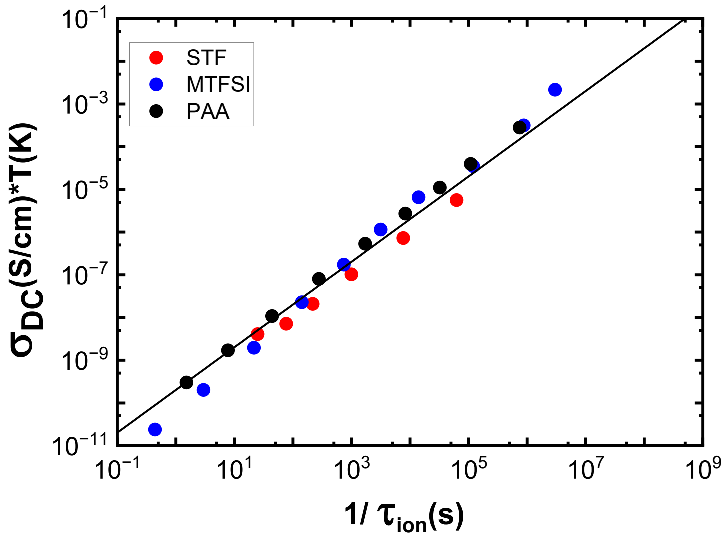

Figure S1. Log-log plot of the experimental DC conductivity multiplied by temperature versus ion relaxation rate for the 3 studied PolyILs discussed in the main text. The black line shows a power law with a slope of unity. Note also the good vertical collapse.

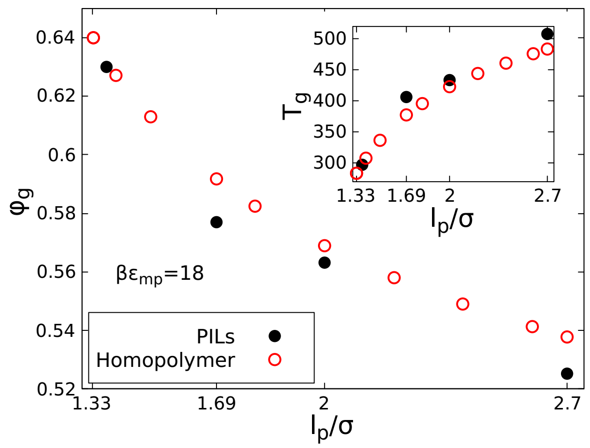

Fig. S2. ECNLE theory[6–13] calculated variation of the glass transition packing fraction based on the criterion of a polymer Kuhn segment activation barrier of 30 kT_g (corresponding to roughly a mean alpha time of $\tau_\alpha(T_g) \approx 25\ s$) as a function of backbone aspect ratio (main) for semiflexible Li-PolyILs and the corresponding results for a reference neutral homopolymer melt. The behavior of PolyILs and homopolymers are very similar, as physically expected since Li is small, strongly decoupled, and induces very little plasticization[13]. The slightly higher T_gs for PolyILs reflects Coulomb modifications of the cage structure as previously discussed[13]. The inset shows the estimated T_g in Kelvin relevant to experiments plotted versus aspect ratio. These results underlie the implementation of Step 1 of the proposed mapping.

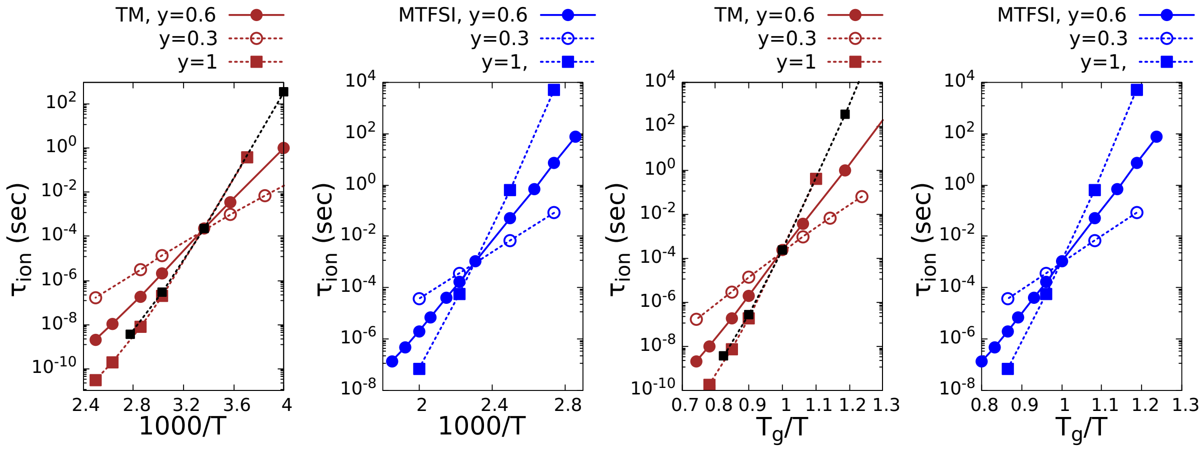

Fig. S3. Left two panels show the predicted temperature dependence of the ion relaxation time of the Theoretical Model (TM) defined in the main text, and MTFSI as a function of inverse temperature (Kelvin) for dielectric constant exponents of y=0.3,0.6,1.0 calibrated at T_g as discussed in the text. The black dashed curve for TM shows the effect of reducing the thermal expansion coefficients by a factor of 2 (in both the liquid and glass states) relative to the baseline values adopted for the y=1 case discussed in the main text. The differences are nearly impossible to see, suggesting the secondary role of the quantitative aspects of the equation of state. The right two figures show the analogous results plotted versus T_g/T. Note for the model adopted the theory predicts nearly apparent Arrhenius temperature dependences, with the exception of the lowest T_g TM model if y=1 is employed.

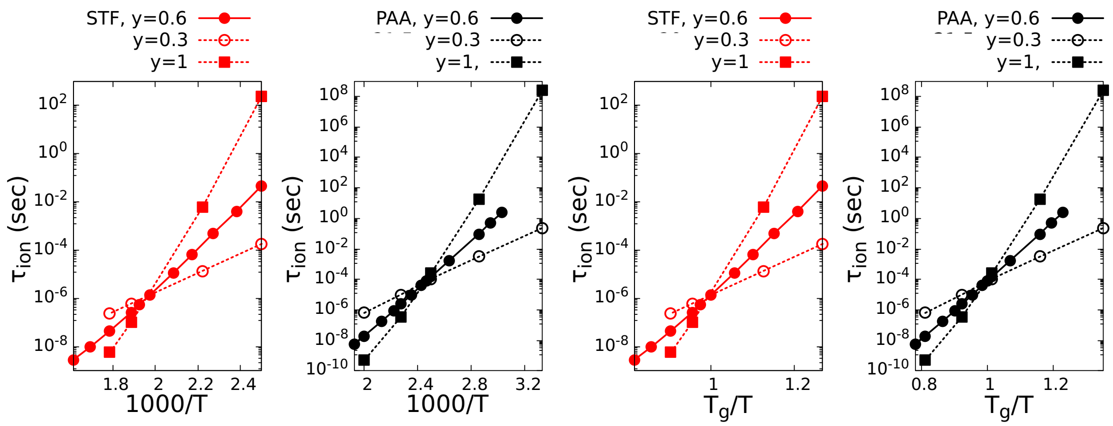

Fig. S4. Results analogous to Fig.S3 for STF and PAA.

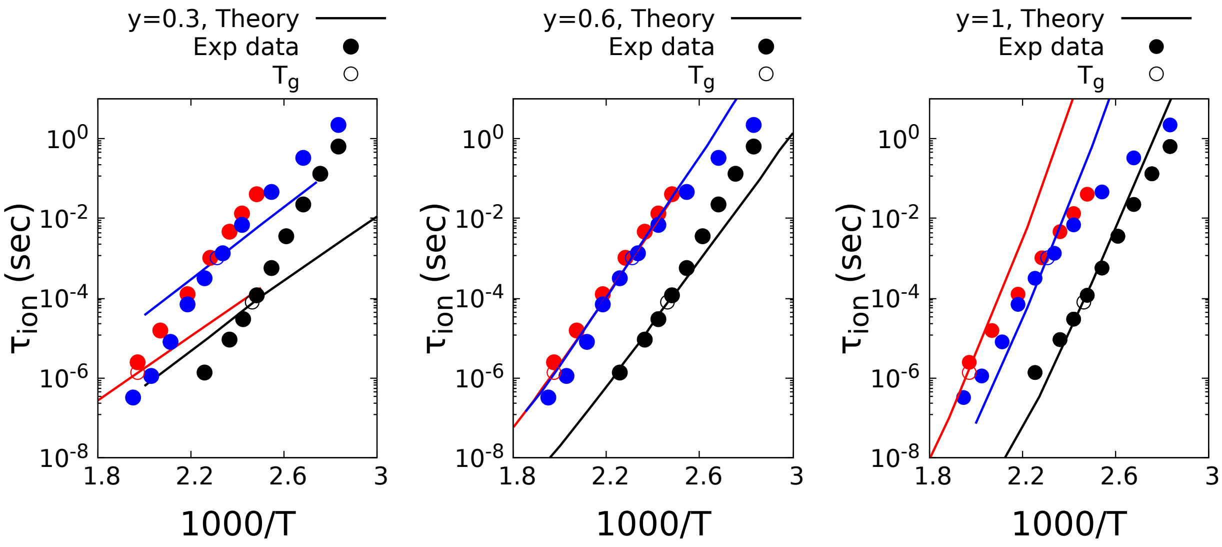

Fig. S5. Direct comparison of experimental ion relaxation time data (solid circles) of STF, MTFSI, and PAA with SCCHT calculations (solid lines) for different dielectric constant exponents of y=0.3 (left panel), y=0.6 (middle panel), and y=1 (right panel) plotted versus inverse temperature (in Kelvin). The values at T_g are shown as open circles. Note the common horizontal and vertical scales. While the y=0.6 calculations capture well the temperature dependence and data collapse of STF and MTFSI, the other y values do not and/or fail to capture the ordering of the Li-ion relaxation times in different polymers (solid lines).

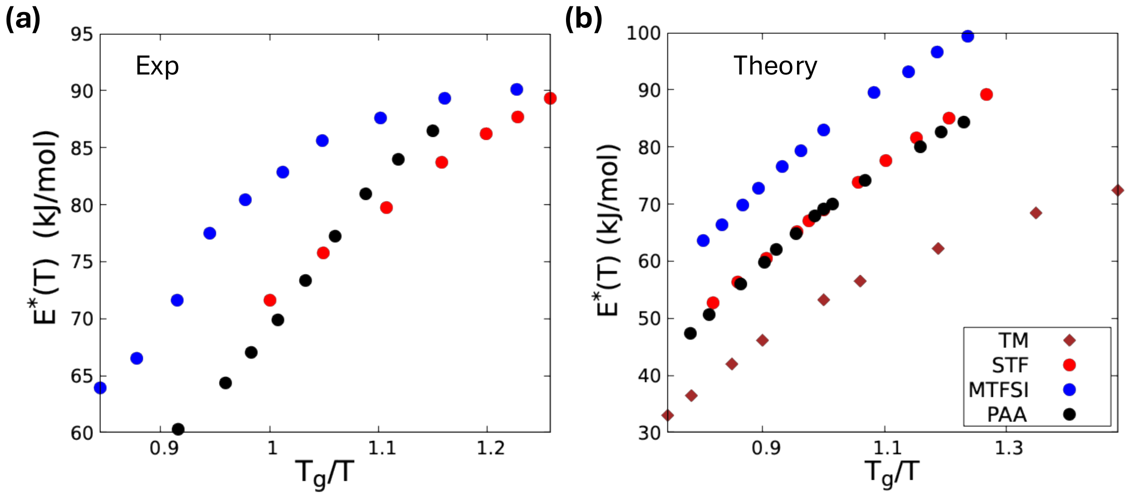

Fig. S6. (a) Experimental (left) and (b) theoretical (right) physical ion barriers $E^*(T)$ in kJ/mole shown in the main text but now plotted as a function of inverse temperature scaled by T_g. Note the horizontal and vertical scales are not identical. Both the theory and experiment $E^*(T)$ data for STF, MTFSI and PAA fall in the window of ~ 60-90 kJ/mole over their common horizontal axis reduced temperature range. The near collapse of the PAA and STF data observed in experiment is consistent with the theoretical results.

Table S1. Results of Steps 1 and 2 of the mapping procedure for the packing fraction, aspect ratio, and dimensionless Coulomb attraction strength at T_g for the studied 3 experimental PolyILs and the theoretical model TM. Right column shows the experimental physical activation barriers in kJ/mole at T_g which by construction of Step 2 of the mapping agrees with the theoretical value; the shown value for the TM is the theoretically predicted value.

PolyIL	φ_g	$T_g(K)$	l_p/σ	$\beta_g \varepsilon_{mp}$	$E^*(T_g)$
STF	0.53	507	2.7	20	71
MTFSI	0.56	433	2.0	22.3	81
PAA	0.58	406	1.69	21.5	69
TM	0.63	297	1.37	21.6	52

References

(1) Bocharova, V.; Wojnarowska, Z.; Cao, P.-F.; Fu, Y.; Kumar, R.; Li, B.; Novikov, V. N.; Zhao, S.; Kisliuk, A.; Saito, T. Influence of Chain Rigidity and Dielectric Constant on the Glass Transition Temperature in Polymerized Ionic Liquids. *J. Phys. Chem. B* **2017**, *121* (51), 11511–11519.

(2) Li, H.; Zhu, Q.; Shinohara, Y.; Wang, Y.; Christakopoulos, P.; Kudlack, A. F.; Huang, Z.; Bonnesen, P. V; Do, C.; Rahman, M. A. Emergent Nanostructure and Ion Transport in Polyzwitterion/Polyanion Blends. *Macromolecules* **2025**, *58* (16), 8658–8669.

(3) M.L. Lehmann; J. Ock; C. Gainaru; Alexei P. Sokolov; X. C. Chen; T. Saito. Single-Ion Conducting Polymer Electrolyte Enabled via Aza-Michael Addition.

(4) Fan, F.; Wang, W.; Holt, A. P.; Feng, H.; Uhrig, D.; Lu, X.; Hong, T.; Wang, Y.; Kang, N.-G.; Mays, J. Effect of Molecular Weight on the Ion Transport Mechanism in Polymerized Ionic Liquids. *Macromolecules* **2016**, *49* (12), 4557–4570.

(5) Marcolongo, A.; Marzari, N. Ionic Correlations and Failure of Nernst-Einstein Relation in Solid-State Electrolytes. *Phys. Rev. Mater.* **2017**, *1* (2), 025402.

(6) Zhou, Y.; Mei, B.; Schweizer, K. S. Activated Relaxation in Supercooled Monodisperse Atomic and Polymeric WCA Fluids: Simulation and ECNLE Theory. *J. Chem. Phys.* **2022**, *156* (11), 114901.

(7) Mirigian, S.; Schweizer, K. S. Unified Theory of Activated Relaxation in Liquids over 14 Decades in Time. *J. Phys. Chem. Lett.* **2013**, *4* (21), 3648–3653.

(8) Mirigian, S.; Schweizer, K. S. Dynamical Theory of Segmental Relaxation and Emergent Elasticity in Supercooled Polymer Melts. *Macromolecules* **2015**, *48* (6), 1901–1913.

(9) Mirigian, S.; Schweizer, K. S. Elastically Cooperative Activated Barrier Hopping Theory of Relaxation in Viscous Fluids. I. General Formulation and Application to Hard Sphere Fluids. *J. Chem. Phys.* **2014**, *140* (19).

(10) Mirigian, S.; Schweizer, K. S. Dynamical Theory of Segmental Relaxation and Emergent Elasticity in Supercooled Polymer Melts. *Macromolecules* **2015**, *48* (6), 1901–1913.

(11) Mirigian, S.; Schweizer, K. S. Elastically Cooperative Activated Barrier Hopping Theory of Relaxation in Viscous Fluids. II. Thermal Liquids. *J. Chem. Phys.* **2014**, *140* (19), 194506.

(12) Mirigian, S.; Schweizer, K. S. Elastically Cooperative Activated Barrier Hopping Theory of Relaxation in Viscous Fluids. II. Thermal Liquids. *J. Chem. Phys.* **2014**, *140* (19).

(13) Das, A.; Rahman, A.; Lehmann, M.; Gainaru, C.; Sokolov, A.; Schweizer, K. S. Theory of Ion-Mediated Segmental Localization, Activated Structural Relaxation, and the Glass Transition in Polymerized Ionic Liquids. *Macromolecules* **2025**, *58* (1), 787–802.
